# Association between depression, happiness, and sleep duration: data from the UAE healthy future pilot study

**DOI:** 10.1186/s40359-022-00940-3

**Published:** 2022-10-21

**Authors:** Mitha Al Balushi, Sara Al Balushi, Syed Javaid, Andrea Leinberger-Jabari, Fatma Al-Maskari, Mohammed Al-Houqani, Ayesha Al Dhaheri, Abdullah Al Nuaimi, Abdullah Al Junaibi, Naima Oumeziane, Marina Kazim, Aisha Al Hamiz, Muna Haji, Ayesha Al Hosani, Leila Abdel Wareth, Wael AlMahmeed, Habiba Alsafar, Fatme AlAnouti, Eiman Al Zaabi, Claire K. Inman, Omar El Shahawy, Michael Weitzman, Ann Marie Schmidt, Scott Sherman, Abdishakur Abdulle, Amar Ahmad, Raghib Ali

**Affiliations:** 1 Public Health Research Center, New York University Abu Dhabi, United Arab Emirates, Abu Dhabi, United Arab Emirates; 2 Department of Psychiatry and Behavioral Sciences, United Arab Emirates University, Al Ain, United Arab Emirates; 3 Institute of public Health, College of Medicine and Health Sciences, United Arab Emirates University, Al Ain, United Arab Emirates; 4 Zayed Military Hospital,, Abu Dhabi, United Arab Emirates; 5 Abu Dhabi Blood Bank Services- Seha, Abu Dhabi, United Arab Emirates; 6 Health and wellness Center, New York University Abu Dhabi, Abu Dhabi, United Arab Emirates; 7 Pathology & Laboratory Medicine Institute, Cleveland Clinic Abu Dhabi, Abu Dhabi, United Arab Emirates; 8 Heart and Vascular Institute, Cleveland Clinic Abu Dhabi, Abu Dhabi, United Arab Emirates; 9 Department of Biomedical Engineering, Khalifa University, Abu Dhabi, United Arab Emirates; 10 College of Natural and Health Sciences, Zayed University, Abu Dhabi, United Arab Emirates; 11 Sheikh Shakhbout Medical City, Abu Dhabi, United Arab Emirates; 12 Department of Population Health, NYU School of Medicine, New York, NY United States of America; 13 MRC Epidemiology Unit, University of Cambridge, Cambridge, UK

**Keywords:** PHQ-8, Depression, Sleep duration, Happiness, Self-reported happiness, Sociodemographic and marital status

## Abstract

**Background:**

The United Arab Emirates Healthy Future Study (UAEHFS) is one of the first large prospective cohort studies and one of the few studies in the region which examines causes and risk factors for chronic diseases among the nationals of the United Arab Emirates (UAE). The aim of this study is to investigate the eight-item Patient Health Questionnaire (PHQ-8) as a screening instrument for depression among the UAEHFS pilot participants.

**Methods:**

The UAEHFS pilot data were analyzed to examine the relationship between the PHQ-8 and possible confounding factors, such as self-reported happiness, and self-reported sleep duration (hours) after adjusting for age, body mass index (BMI), and gender.

**Results:**

Out of 517 participants who met the inclusion criteria, 487 (94.2%) participants filled out the questionnaire and were included in the statistical analysis using 100 multiple imputations. 231 (44.7%) were included in the primary statistical analysis after omitting the missing values. Participants’ median age was 32.0 years (Interquartile Range: 24.0, 39.0). In total, 22 (9.5%) of the participant reported depression. Females have shown significantly higher odds of reporting depression than males with an odds ratio = 3.2 (95% CI:1.17, 8.88), and there were approximately 5-fold higher odds of reporting depression for unhappy than for happy individuals. For one interquartile-range increase in age and BMI, the odds ratio of reporting depression was 0.34 (95% CI: 0.1, 1.0) and 1.8 (95% CI: 0.97, 3.32) respectively.

**Conclusion:**

Females are more likely to report depression compared to males. Increasing age may decrease the risk of reporting depression. Unhappy individuals have approximately 5-fold higher odds of reporting depression compared to happy individuals. A higher BMI was associated with a higher risk of reporting depression. In a sensitivity analysis, individuals who reported less than 6 h of sleep per 24 h were more likely to report depression than those who reported 7 h of sleep.

**Supplementary Information:**

The online version contains supplementary material available at 10.1186/s40359-022-00940-3.

## Introduction

Depression is defined as a set of disorders ranging from mild to moderate to severe [1]. It is widely recognized as a major public health problem worldwide [2]. Reports from the Global Burden of Diseases declared major depressive disorder as one of the top three causes of disability-adjusted life years [3, 4]. The effect of depression has been extensively studied on the individual’s daily functioning and productivity. This is directly reflected in increased economic costs per capita [3, 4]. For example, in Catalonia in 2006, the average annual cost of an adult with depression was close to 1800 Euros, and the total annual cost of depression was 735.4 million Euros. These costs were linked directly to primary care, mental health specialized care, hospitalization, and pharmacological care, as well as indirect costs due to productivity loss, temporary and permanent disability [4].

To measure the level of depression in non-clinical populations; clinical and epidemiological studies have often used the established and validated eight-item Patient Health Questionnaire scale (PHQ-8) instead of the nine- item Patient Health Questionnaire scale (PHQ-9) [5]. As has been confirmed by previous studies, the scale can detect major depression with sensitivity and a specificity of 88%, to classify subjects into depressed or non-depressed, respectively [6–8]. Regionally, in Jordan, Lebanon, Syria and Afghanistan, the cutoff point of 10 was used [9–12]. Similarly, in UAE, studies have mostly used the cutoff point of 10 [13–16].

Recent studies have focused on exploring the methods of improving individual and environmental effects on disability related to depression by investigating its triggers and associations [2, 10]. Factors such as sleep duration and self-reported happiness are evidenced to be predictors for depression [17, 18]. Sleep duration can be considered a risk factor for lower well-being [19]. The number of sleep hours has also been found to have a causal relationship with depression as well as self-reported happiness [20]. For example, shorter sleep duration might lead to lower positive emotions such as self-reported happiness and showed stronger associations with negative emotional affect [21, 22]. One question that needs to be asked, however, is the nature and strength of the association between these three variables respectively.

Sleep duration is determined by how many hours an individual sleeps over 24 h. Individuals with depression often have poor sleep status including abnormal REM (rapid eye movement), and insomnia (difficulty falling asleep or staying asleep). Abnormal REM may contribute to the development of altered emotional processing in depression [23]. It was reported that people with insomnia might have a ten-fold higher risk of developing depression in contrast to people who get a good night’s sleep [24]. Also, 75% of depressed individuals will have trouble falling asleep or staying asleep [25]. A bidirectional relationship between sleep duration and reported depression has been investigated [24, 26]. Such studies are unsatisfactory because they do not explore the association between major depressive disorder and sleep duration based on specific population demographics. In this study we are considering other sociodemographic factors such as age, gender, and marital status.

Interest in studying happiness in the context of mental health status (such as depression and anxiety) has been growing recently [27]. The findings of some research papers suggest that self-reported happiness is a potential factor in the prevention and management of depression [27, 34]. Happiness is correlated to a person’s ability to approach situations in a less stressful manner and to an individual’s capacity to perceive and control their own feelings. This indicates that higher happiness levels may have a protective effect on depression [27]. Well-being has been defined as the combination of feeling good and functioning well [28]; conversely, quality of life could be defined as an individual’s satisfaction with his or her actual life compared with his or her ideal life. Evaluation of the quality of life depends on one’s value system [29].

Moreover, studies have shown that individuals use various chronically accessible and stable sources of information when making life satisfaction judgments [30]. Yet, well-being, quality of life and life satisfaction are multidimensional constructs which include complex cognitive evaluation processes and cannot be adequately assessed by using a single item or question [31]. Unlike quality of life, which typically requires detailed assessments to ascertain [32], happiness is easier to evaluate using a single item question [33]. Investigating the association between happiness and depression would add valuable information to public health research as the relationship between happiness and depression is observed to be bidirectional (i.e., one variable can predict the other, and people might not report depression but are more likely to report feeling unhappy) [34].

In addition, there are some factors which confound with sleep duration and self-reported happiness, such as age, gender, and marital status [36, 37]. For example, reviews reported that a prognosis of depression was improved with increasing age [38]. Conversely, another study showed that older age was associated with worsening of depressive symptoms [37]. Moreover, depressive symptoms could worsen among widowed individuals as their age increased [40]. Some research findings reflected that females are more likely to perceive depression compared to males [38, 41]. Other studies showed significant interplay between marital status and depressive symptoms [40, 42]. For example, being unmarried could lead to perceiving and developing depressive symptoms [43], while a worsening in depression symptoms among married individuals could lead to separation or being unmarried [42, 44]. So, further exploration is required to study the interplay of these variables together.

The United Arab Emirates (UAE) is a high-income developed country which has undergone a rapid epidemiological transition from a traditional semi-nomadic society to a modern affluent society with a lifestyle characterized by over-consumption of energy-dense foods and low physical activity [47]. The UAE was ranked 15th out of 157 countries included in the WHO World Happiness Report with a score of 7.06 [48]. Despite this, depression has been identified as the third leading cause of disability in the UAE [3]. Nevertheless, there are few studies in the Gulf region examining the relationship between happiness, sleep duration and depression [36, 49, 50]. Therefore, studying the association between depression, happiness, and sleep in the United Arab Emirates (UAE) population would be of interest to the public health field in this part of the world. This study aimed to examine the relationship between depression, self-reported happiness, and self-reported sleep duration after adjusting for age and gender using the UAE Healthy Future Study (UAEHFS) pilot data.

## Materials and methods

### Study design

This was a pilot prospective cohort study conducted from January 2015 to May 2015. The participants were recruited from two health care centers in Abu Dhabi. Participants completed an online questionnaire including questions on demographic data, PHQ-8 score, self-reported sleep duration, and self-reported happiness score. Physical measurements such as Body Mass Index (BMI) were collected during the recruitment visit.

### Participants’ eligibility criteria and recruitment

Seven hundred and sixty-nine UAE nationals aged ≥ 18 years were invited to participate voluntarily in the pilot study. Volunteers from the general population with inclusion criteria of age 18 or greater; able to consent; UAE nationals, resident in Abu Dhabi Emirate. All potential participants were given participant information leaflets in either Arabic or English to read and had the opportunity to ask questions prior to completion of the recruitment process. Participants signed an informed consent and were asked to complete a detailed questionnaire. However, 243 invited subjects did not respond, and their reasons for not participating were recorded [45]. Out of 517 participants who met the inclusion criteria, 487 (94.2%) participants filled out the questionnaire and were included in the statistical analysis [47].

## Measures

The PHQ-8 questionnaire was used to measure the participants’ depression levels [6, 39, 51]. This study used the cutoff point of 10 as well based on the common local practice considering that there are no specific related research guidelines in UAE [13–16]. The PHQ-8 score was dichotomized into no-depression (total PHQ-8 < 10) versus depression (≥ 10) [6, 51].

Happiness was measured using a one-question item that asked participants, “In general, how happy you are?’’ Those who responded as extremely happy, very happy and moderately happy were grouped as “happy” while those responding as moderately unhappy, very unhappy, and extremely unhappy were grouped as “unhappy”.

Demographic variables such as age, gender, and marital status (single, married, and others) were also included in the reference. Sleep duration data was collected as an ordinal variable (number of hours) by asking how many hours of sleep the participant gets in a 24-hour period, including naps. Sleep duration was categorized into five categories (see Table 1) to avoid the linearity assumption between sleep duration and depression status as well as to be able to compare average sleepers to shorter and longer sleepers. The questionnaire was translated from English into Arabic and back-translated into English to check for linguistic validity.

**Table 1:** *Number (percentages) of the analyzed variables and median (IQR) for Age and BMI.*

Variable	Group	PHQ-8 < 10	PHQ-8 ≥ 10	P-value
Gender	Female	62 (83.8)	12 (16.2)	0.028^a^
	Male	147 (93.6)	10 (6.4)	
Sleep (hours)	< 6	39 (83)	8 (17)	0.284^a^
	= 6	53 (93)	4 (7)	
	= 7	57 (95)	3 (5)	
	= 8	44 (89.8)	5 (10.2)	
	> 8	16 (88.9)	2 (11.1)	
Marital Status	Single	120 (93)	9 (7)	0.205^a^
	Married	15 (93.8)	1 (6.2)	
	Other	74 (86)	12 (14)	
Happiness	Happy	204 (91.1)	20 (8.9)	0.136^a^
	Unhappy	5 (71.4)	2 (28.6)	
Total		209 (90.5)	22 (9.5)	
		Median (IQR)	Median (IQR)	P-value
Age	years	33.0 (25.0, 40.0)	24.5 (22.0, 35.0)	0.012^b^
BMI	kg/m^2^	27.6 (23.4, 31.2)	28.8 (24.2, 33.0)	0.444^b^

Note: ^a^Fisher’s exact test p-values for categorical data and ^b^Wilcoxon rank sum test for continuous data

Body mass index (BMI) was obtained via physical measurement using Tanita MC-780 MA Segmental Body Composition Analyzer [52]. All physical measurements were collected by a clinical research nurse. Additional details of the study recruitment have been previously described [47].

## Statistical analysis

All eligible participants 487 (94.2%) were included in a sensitivity analysis (using 100 multiple imputations). After excluding participants with missing values, 231 (44.7%) were included in the primary statistical analysis. The PHQ-8 questionnaire was used in this statistical analysis with two additional possible options to select for each question (i.e., P2A – P2H). These were “Do not know (UN)” and “Prefer not to answer (DA)”, which were treated as missing values in the statistical analysis. Fisher’s exact test was used to investigate the association between depression and categorical variables, such as Sleep (hours), Happiness, Marital Status, and Gender. Wilcoxon rank-sum test was performed to investigate differences in the distribution of age and BMI within the depressed versus non-depressed group respectively.

To examine the factors associated with depression, a multivariate logistic regression model was performed with the dichotomized PHQ-8 score as the outcome. The predictors were Happiness score (Happy vs. Unhappy), Age (linear), Gender (Females vs. Males), BMI (linear), and Marital status (categorical). Interquartile-range odds ratios (IQR-OR’s) for continuous predictors and simple odds ratios (OR’s) for categorical predictors with 95% confidence intervals (Cis) were estimated for continuous and categorical variables respectively; corresponding p-values were calculated [53].

In sensitivity analysis, a multivariate logistic regression model and a multivariate linear regression model were conducted using multiple imputations (see sensitivity analysis section). All applied statistical tests were two-sided; p < 0.05 were considered statistically significant. No adjustment for multiple comparisons was made. Statistical analyses were performed in R version 4.0.2 [54].

## Sensitivity analysis

The primary statistical analysis included subjects with at least one non-missing value. However, in a sensitivity analysis, a multivariate imputation by chained equations (MICE) procedure was applied with Classification and Regression Trees (CART) to impute missing values [55]. 100 multiple imputations were used [56]. Rubin’s rules were used to combine the multiple imputed estimates [57].

The pattern of missing values was investigated, and it was found that subjects who “did not want to answer” were not systematically different from those who answered the questionnaire. Therefore, “prefer not to answer” was recorded as missing value in the statistical analysis and was considered a missing variable in the sensitivity analysis [55].

## Results

**Fig. 1:**
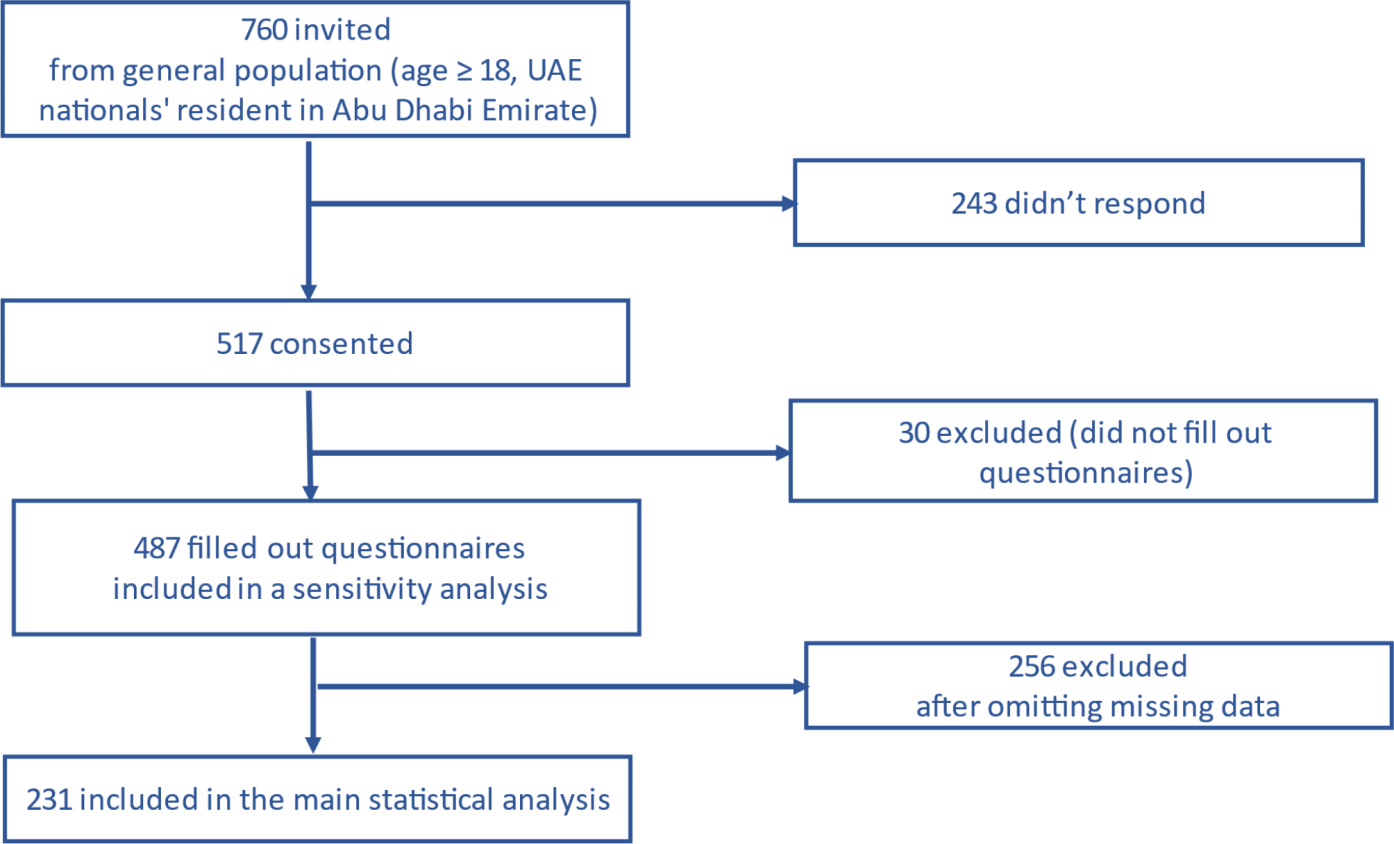
Flow chart of UAEHF pilot study (describes all the key phases from the recruitment up to the inclusion in the study analysis)

Note: This Figure represents the data of the included participants in the main statistical analysis after omitting missing values.

Of 517 participants who consented to participate in the UAEHF pilot study, 487 (94.2%) had completed questionnaire data [47]. Figure 1 describes all key phases from recruitment up to inclusion in the study and in the statistical analysis. After omitting missing values, 231 (44.7%) participants were included in the main statistical analysis. However, 487 (94.2%) participants were included in the sensitivity analysis (using 100 multiple imputations) [73]. The median age of the UAEHFS pilot data participants was 32.0 years (Interquartile Range: 24.0–39.0). The percentage of females included in the study was 32%, which represented the UAE population well [59]. Therefore, we did not make any adjustments for gender bias.

The number of observed values (%) of each categorical variable was presented by the PHQ-8 categories in Table 1, where the majority of the study participants (90.5%) had PHQ-8 score less than 10. When categorical groups were compared within the PHQ-8 depression group (< 10 versus ≥ 10), there was only a statistically significant difference between females versus males with a Fisher exact p-value = 0.028. Table 1 shows that there was a statistically significant difference in the age distribution across the depression groups (p-value = 0.012). There was no statistically significant difference in the BMI measurements between the PHQ-8 groups (p-value = 0.44).

The result of the primary analysis showed that females have statistically significantly greater odds of reporting depression compared to males OR = 3.2 (95% CI:1.1, 8.9), p-value = 0.024 (Table 2). Furthermore, there was an approximately 5-fold greater odds of reporting depression for unhappy than for happy individuals. However, this was not statistically significant (p-value = 0.10). Similarly, the sleep duration and marital status were both not statistically significant (p-value of 0.284 and 0.205, respectively; Table 2). However, in a sensitivity analysis, people who reported sleep duration of less than 6 h were more likely to report depression as compared to the people who reported sleep of 7 h OR (95% CI) of 2.6 (1.0, 6.4), p-value = 0.040 and 1.136 (1.015, 1.272), p-value = 0.027.

**Table 2:** *The results of the primary analysis of the fitted multivariate logistic regression model*

Variable	OR (95% CI)	P^b^
Happiness - Happy	Reference	
Happiness - Unhappy	5.5 (0.7, 42.2)	0.100
Age (linear)	0.3 (0.1, 1.0)^a^	0.056
BMI (linear)	1.8 (1.0, 3.3)^a^	0.064
Gender = Males	Reference	
Gender = Females	3.2 (1.2, 8.9)	0.024
Sleep = 7	Reference	
Sleep < 6	4.0 (0.9, 17.4)	0.061
Sleep = 6	1.3 (0.2, 6.9)	0.775
Sleep = 8	1.5 (0.3, 7.3)	0.585
Sleep > 8	1.2 (0.2, 9.2)	0.848
Married	Reference	
Single	0.9 (0.3, 3.0)	0.8
Others	0.2 (0.02, 3.3)	0.3

Note: ^a^Interquartile-range odds ratio for age and BMI, which compares the 3rd quartile with the 1st quartile, and simple odds ratios for the categorical predictors (happiness, gender, sleep and marital status) compare each group with the reference group (the largest group) with corresponding 95% confidence interval (95% CI). ^b^Wald’s p-values are presented for each variable

To compute Interquartile Range (IQR) odds ratios for continuous variables, the age and BMI variables were divided by their IQR values of 15 and 8.7, respectively [54, 56]. Table 2 shows that for one interquartile-range increase in the age and BMI, the IQR-OR was 0.34 (95% CI: 0.12, 1.0) and 1.8 (95% CI: 0.97, 3.3), respectively, where both results were statistically significant with a p-value of 0.056 and 0.064 individually.

The results of the sensitivity analysis using 100 multiple imputations (Table 3) were approximately similar to the result of the multivariate logistic ordinal regression analyses using omitted data (Table 2). However, the happiness variable was statistically significant OR = 5.1 (95%CI: 1.7, 15.7, p-value = 0.005), and there was a statistically significant difference between the sleep variable for less than six hours as compared with seven hours OR = 2.6 (95% CI: 1.0, 6.4, p-value = 0.04). Supplementary Fig. 1 illustrates the percentages of missing values in the variables included in this statistical analysis. Marital status had the lowest number of missing values (5.3%), followed by BMI (12.7%) and the Sleep variable with 12.9% missing values. The Happiness variable had 21.1% missing values. The PHQ-8 variables had 32.6–35.1% missing values. Supplementary Table 1 shows the percentages of the “Prefer not to answer (DA)” and “Do not know (UN)” in the eight PHQ questions, where the percentages vary between 16 and 18.5% between the eight questions. The percentages of “Prefer not to answer (DA)” and “Do not know (UN)” in the happiness variable was 2.5% and 2.3%, respectively, which were also considered missing values, although these were not missing at random and can be correlated with one of the PHQ-8 answers. However, this has been addressed in the multiple imputation analysis.

**Table 3:** *Results of the sensitivity analysis using multivariate logistic regression models with a sample size N = 487*

Variable	OR (95% CI)	P^b^
Happiness – Happy	Reference	
Happiness - Unhappy	5.1 (1.7, 15.7)	0.004
Age (linear)	0.5 (0.3, 1.0)^a^	0.060
BMI (linear)	1.7 (1.1, 2.5)^a^	0.021
Gender = Males	Reference	
Gender = Females	1.6 (0.9, 3.0)	0.154
Sleep = 7	Reference	
Sleep < 6	2.6 (1.0, 6.4)	0.040
Sleep = 6	0.9 (0.3, 2.6)	0.887
Sleep = 8	0.9 (0.3, 2.4)	0.779
Sleep > 8	1.1 (0.3, 3.7)	0.853
Married	Reference	
Single	0.9 (0.4, 2.1)	0.885
Others	0.7 (0.2, 2.9)	0.597

Note: ^a^Interquartile-range odds ratio for Age and BMI, which compares the 3rd quartile with the 1st quartile, and simple odds ratios for the categorical predictors (happiness, gender, sleep and marital status) compare each group with the reference group (the largest group) with corresponding 95% confidence interval (95% CI). ^b^Wald’s p-values are presented for each variable. Results were summarized using Rubin’s rules

An additional sensitivity analysis was performed using the ordinal PHQ-8 score as an outcome in a multivariate linear regression model (see supplementary Table 1). The result of this sensitivity analysis was very similar to the sensitivity analysis in Table 3.

## Discussion

Overall, there is a lack of quantitative research examining the relationship between depression, perceived happiness, and sleep duration and other confounding factors in the Gulf region [35,46, 36, 49, 60]. The UAE Healthy Future Study is the first prospective cohort study of the UAE population and one of the few studies in the region which examines such relationships between happiness, sleep duration and quality, and depression using PHQ-8. The evidence collected from this study confirms what has been published in the literature. For instance, in this study, it has been found that males were less likely to report depression symptoms than females, which is similar to what has been documented by other studies [38, 41]; where women reported more depressive symptoms than men [60]. In addition, the results of this study revealed that older people have a lower odds of reporting depression as compared with younger people, suggesting a possible protective age effect.

This study has used the pilot data of the UAE Healthy Future Study, as recruitment into the main cohort study is still ongoing. However, we plan to use the main UAEHFS data to confirm the results and provide further understanding of these findings.

The association between sleep duration and depression has been intensively studied in the literature [23, 24]. The findings of this study revealed that individuals who reported less than 6 h of sleep per 24 h were more likely to report being depressed compared to those who reported 7 h of sleep. This is similar to what has been reported in the literature that participants who reported insufficient sleep showed a 62–179% increase in the prevalence of depression versus those sleeping 6 to 8 h per day and reporting sufficient sleep (P < 0.05) [61, 62].

Furthermore, self-reported happiness has been identified as a potential protective and management factor for depression [27]. The result of this study shows that unhappy individuals have approximately 5-fold higher odds of reporting depression compared to happy individuals, and this aligns with what has been found in the literature [27].

Additionally, a small number of studies found that insufficient sleep is associated with lower happiness in healthy adults using a self-reported questionnaire as a single item for measuring happiness [22]. Moreover, some longitudinal studies have found that the next-day happiness is lowered following a shorter sleep duration [63]. However, the associations between sleep and happiness have not been well-explored in adults. Our findings will add to the available evidence and will help to bring novel data from the Gulf Cooperation Council (GCC) countries about the association between sleep duration, self-reported happiness, and depression.

A further finding is that marital status can contribute to health and self-reported happiness in a bidirectional way [49]. Several studies have found that there is an association between depression and marital status [63, 64]. There is an increased risk of reporting depression for divorced and separated people. It is frequently asserted that marriage is more beneficial for the mental health of men than women, but the evidence for this is far from clear-cut [65]. Single people have a higher level of depression as compared to married people and some studies have found that married people have a better mood than single people considering factors of age, gender, and education level [37, 40, 41, 66]. Research does not yet clarify whether gender differences in the prevalence of anxiety-mood disorders are greater among the married than the never-married or the previously married [65]. However, the mechanisms underlying the relationship between depression and marital status are not yet entirely clear and require further exploration.

An association has been reported between BMI categories and depression [67, 68]. Higher BMI is a risk factor for the likelihood of developing depression in individuals [69]. Underweight increases the risk of depression as well [70]. Our study considered the BMI factor in the data analysis of this study. This statistical analysis shows that as BMI increases, the odds of reporting depression also increase. This result is comparable to other research finding that participants with central obesity had an increased chance of depression [70,71,72].

The UAEHFS is a unique cohort study in the UAE and Gulf region as it allows researchers to investigate the association between disease outcomes and related risk factors [58]. In this study, we investigated the association between depression and sleep duration, self-reported happiness, BMI, and sociodemographic status using the UAEHFS pilot data, which presents a population that has not been studied. The result of our study needs to be confirmed in the main UAEHFS data.

## Strengths and Limitations

Missing values were omitted in the primary statistical analysis, which decreased the sample size and can lead to overfitting in the main finding [55]. Therefore, the number of participants with the PHQ-8 ≥ 10 (i.e. – events) was 22 which is a limitation in this data set because it does not allow us to fit a complex multivariate statistical regression model if we would follow the statistical rule of thumb “ten events per predictor” [56, 57, 73]. Thus, sensitivity analysis was performed, and the result of the sensitivity analysis (Table 3) was approximately the same as the main finding (Table 2).

Although some limitations were found in this study, the findings provide future direction to mental health research. Further investigation is needed with a larger sample size using the main UAEHFS data to have a better picture of the role of happiness, marital status, sleep, and social demographic variables in association with depression and mental health disorders.

## Conclusion

The results of this study indicate that females are more likely to report depression compared to males. Older age is associated with a decrease in self-reported depression. Unhappy individuals are approximately 5-fold higher odds to report depression compared to happy individuals. BMI was positively associated with reporting depression. The result of the sensitivity analysis shows that individuals who sleep less than 6 h per day are more likely to report depression compared to those who sleep 7 h per day.

The results of this study have a potential value for researchers and public health professionals as it presents novel data on the PHQ-8 score in a healthy UAE population, which has not been explored before. Furthermore, the results of this study can help contribute to the knowledge base on current and potential population mental health impact in the UAE and Gulf Region.

## Electronic supplementary material

Below is the link to the electronic supplementary material.

Supplementary Material 1

## Data Availability

Data are from the United Arab Emirates Healthy Futures (UAEHFS) study. A de-identified data set can be shared subject to the policies of the approving ethics committees and the data access policy of the UAEHFS. The New York University Abu Dhabi IRB approved informed consent form described how participant data would be shared with other researchers. The consent form states that researchers who are interested in accessing study data will contact the data access/ethics committee to be granted access to the data. Once approved, de-identified data can be made available. Researchers who meet the criteria for access to confidential data may contact the IRB at IRBnyuad@nyu.edu to gain access to the data.

## Declarations

Please address all correspondence concerning this manuscript to me at ma4643@nyu.edu.
