# Supplementary material for: Association between depression, happiness, and sleep duration: data from the UAE healthy future pilot study
Source: BMC Psychol. 2022 Oct 21;10:235. doi: 10.1186/s40359-022-00940-3 (PMC9587590; doi:10.1186/s40359-022-00940-3)
Supplement: Supplementary file 1 — Supplementary Material 1 [file 40359_2022_940_MOESM1_ESM.docx]

**Supplementary table 1**

The percentages of the “Prefer not to answer (DA)” and “Do not know (UN)” in the eight PHQ questions.

|  | **P2A** | **P2B** | **P2C** | **P2D** | **P2E** | **P2F** | **P2G** | **P2H** |
| --- | --- | --- | --- | --- | --- | --- | --- | --- |
| **DA** | 3.90% | 3.10% | 3.30% | 3.30% | 3.10% | 4.50% | 4.10% | 4.10% |
| **UN** | 12.30% | 13.80% | 14.80% | 12.70% | 12.70% | 14.20% | 13.30% | 14.40% |

**Supplementary Figure 1**

The percentages of missing values by each variable included in this statistical analysis.


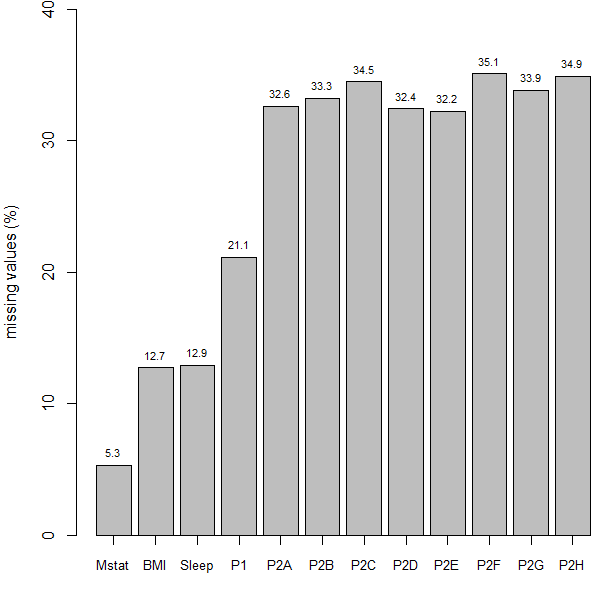


Higher percentages of missing values are observed by the PHQ-8 score variables as compared with sleep, marital status (Mstat), and the happiness (P1) variables respectively.
